# Photodynamic Therapy with 5-Aminolevulinic Acid Versus Topical Corticosteroids in the Treatment of Oral Lichen Planus: A Randomized Clinical Trial with Lesion Site-Specific Analysis

**DOI:** 10.3390/pharmaceutics17111381

**Published:** 2025-10-24

**Authors:** Aleksandra Pietruska, Magdalena Sulewska, Patryk Wiśniewski, Jagoda Tomaszuk, Emilia Szymańska, Katarzyna Winnicka, Joanna Narolewska, Małgorzata Pietruska

**Affiliations:** 1 Student’s Research Group, Department of Periodontal and Oral Mucosa Diseases, Medical University of Bialystok, ul. Waszyngtona 13, 15-269 Bialystok, Poland; perio@umb.edu.pl (A.P.); magdalena.sulewska@umb.edu.pl (M.S.); patryk.wisniewski@umb.edu.pl (P.W.); jagoda.tomaszuk@umb.edu.pl (J.T.); 2 Department of Pharmaceutical Technology, Medical University of Białystok, Mickiewicza 2c, 15-222 Białystok, Poland; emilia.szymanska@umb.edu.pl (E.S.); katarzyna.winnicka@umb.edu.pl (K.W.); 3 Symmetry Medical Center, ul. Św. Rocha 5, 15-879 Białystok, Poland; askanarolewska@gmail.com

**Keywords:** oral lichen planus, photodynamic therapy, 5-aminolevulinic acid, topical corticosteroids, ALA-PDT, randomized clinical trial, REU index, visual analog scale

## Abstract

**Objective**: Oral lichen planus (OLP) is a chronic immune-mediated condition of the oral mucosa, commonly associated with pain and burning sensations that impair quality of life. This study aimed to compare the efficacy of photodynamic therapy with 5-aminolevulinic acid (ALA-PDT) and topical glucocorticosteroids (CT) in the treatment of OLP, considering lesion location on keratinized and non-keratinized mucosa. **Materials and Methods**: A randomized clinical trial was conducted on 90 patients with histologically confirmed OLP. Participants were allocated to receive either ALA-PDT in addition to novel oromucosal emulgel containing 5% ALA (five weekly sessions) or clobetasol propionate applied twice daily for two weeks. Lesion area, clinical severity (Reticulation, Erythema, Ulceration—REU index), and subjective symptoms (Visual Analog Scale—VAS) were evaluated before treatment, immediately after, and six months after therapy. **Results**: ALA-PDT achieved significantly greater and more durable reductions in lesion area, REU scores, and VAS values compared to CT, particularly on non-keratinized mucosa (mean lesion reduction from 2.64 to 0.56 cm^2^ at six months; *p* < 0.0001). CT therapy showed initial improvement but was followed by relapse at six months. Both treatments were well tolerated, with only mild transient adverse effects reported. **Conclusions**: ALA-PDT, especially when applied to non-keratinized oral mucosa, provides superior and longer-lasting therapeutic outcomes than topical CT. The application of novel ALA-loaded emulgel enhances treatment efficacy and tolerability, supporting PDT as a promising alternative for OLP management.

## 1. Introduction

Oral lichen planus (OLP) is a chronic, immune-mediated disease characterized by burning sensations, pain, and hypersensitivity, significantly impairing patients’ quality of life [1,2]. OLP is a relatively common lesion affecting the oral mucosa. Recent meta-analyses estimate its prevalence at approximately 0.89–1.01%. Statistically, the condition occurs more frequently in women and individuals over the age of 40. A higher prevalence has been reported in South America, followed by Africa and Europe, with the lowest rates observed in Asia [3,4].

Oral lesions may present as solitary manifestations or occur simultaneously in multiple locations. They are most commonly found on the buccal mucosa and the lateral surfaces of the tongue. Less frequently, lesions are observed on the gingiva, lips, palate, retromolar trigone area, and the floor of the mouth [4].

Although the clinical presentation of OLP has been well characterized, its etiology and pathogenesis remain incompletely understood. Substantial evidence supports the hypothesis that immune dysregulation—particularly cytotoxic T-cell-mediated responses—plays a central role in the development of the disease. This aberrant immune activity results in the destruction of basal keratinocytes, ultimately leading to the formation of lesions on the skin and oral mucosa. Both genetic susceptibility and environmental factors are believed to contribute to disease onset and progression [5].

Genetic studies have identified several immune-related genes that may predispose individuals to OLP, particularly those encoding proteins of the major histocompatibility complex (MHC), which are essential for antigen presentation and modulation of immune responses [6]. Additionally, polymorphisms in genes responsible for pro-inflammatory cytokines, such as tumor necrosis factor-alpha (TNF-α) and interleukin-6 (IL-6), have been associated with heightened inflammatory responses and increased T-cell activation, both of which are implicated in OLP pathogenesis [7]. While genetic variability likely plays a role in disease susceptibility, the underlying molecular mechanisms have yet to be fully elucidated.

A broad range of environmental triggers has also been implicated in the development of OLP. These include viral infections—most notably hepatitis C virus—as well as bacterial and fungal pathogens, various pharmacological agents, radiation exposure, psychological stress, systemic diseases (such as thyroid disorders or ulcerative colitis), depression, and exposure to dental amalgam fillings [8,9,10,11,12,13,14].

In recent years, oxidative stress (OS) has emerged as an important factor in the pathophysiology of OLP. Oxidative stress is defined as a disruption of the balance between pro-oxidants and antioxidants, resulting in altered redox signaling and homeostasis, and leading to molecular and cellular damage [15]. In the context of OLP, activated T lymphocytes stimulate the production of pro-inflammatory cytokines, which promote the generation of reactive oxygen species (ROS). These ROS can damage various cellular structures, inducing apoptosis of keratinocytes. Elevated levels of keratinocyte apoptosis have been consistently observed in OLP lesions, supporting the role of oxidative stress in disease development [16,17].

Furthermore, Hassan et al. [18] reported that oxidative stress leads to the accumulation of hydrogen peroxide (H_2_O_2_), which induces vacuolar degeneration within the basal epithelial layer. This mechanism may contribute directly to the histopathological features of OLP [18].

In summary, current evidence suggests that the initiation of OLP involves the presentation of unidentified antigens to CD4+ and CD8+ T lymphocytes. The subsequent activation of helper T cells leads to the proliferation and cytotoxic activity of CD8+ T cells directed against basal keratinocytes. This immune-mediated process ultimately results in keratinocyte apoptosis and disruption of the basement membrane structure, which are hallmark features of the disease [19,20].

Treatment of OLP remains challenging due to the recurrent nature of lesions and the absence of a causal therapy [20,21]. Topical corticosteroids (CTs) are considered the gold standard of care; however, long-term use carries the risk of adverse effects, including mucosal atrophy and fungal infections [20,22].

Among topical agents, 0.05% clobetasol propionate is most commonly employed. Other corticosteroids including triamcinolone, betamethasone, fluocinonide, fluticasone, dexamethasone, and prednisolone are also used in various formulations—such as ointments, oral suspensions, aqueous solutions, mouth rinses, and adhesive pastes—and have demonstrated both efficacy and safety in clinical practice [23,24]. A recent randomized, phase II clinical trial evaluated a novel mucoadhesive clobetasol patch (Rivelin^®^-CLO) in patients with erosive OLP. The study reported significant improvements in both subjective and objective symptoms in the treatment group compared to placebo, with a favorable safety profile [25].

Intravenous corticosteroid injections, including triamcinolone acetonide, hydrocortisone, dexamethasone, and methylprednisolone, have also demonstrated efficacy in erosive OLP. However, this modality is associated with considerable patient discomfort, and only a limited number of erosions can be treated during a single session [25,26]. Therefore, in cases of multifocal OLP, alternative and relatively safer treatment options such as photodynamic therapy (PDT) should be considered.

For refractory OLP, systemic corticosteroids—such as dexamethasone or prednisone—are commonly prescribed. Oral prednisone is typically administered at a dose of 0.5 mg/kg for 4–6 weeks [27]. Nevertheless, prolonged systemic corticosteroid therapy may lead to significant adverse effects, including muscle weakness, sleep disturbances, weight gain, pathological fractures, anemia, acne, striae, and menstrual irregularities [28]. To overcome or minimize these side effects, oral mini-pulse therapy has been proposed as a novel treatment approach [29]. Malhotra et al. [29] compared a mini-pulse regimen of 5 mg oral betamethasone administered two consecutive days per week with 0.1% triamcinolone acetonide paste in patients with OLP. The study demonstrated comparable clinical responses in both groups; however, earlier clinical improvement was observed in the oral betamethasone group. Reported adverse effects, including facial edema, headache, and muscle weakness, were mild, transient, and reversible [29].

The meta-analysis by He et al. [30] and a systematic review by Akram et al. [31] demonstrate that PDT is comparably effective to topical corticosteroids in managing OLP, particularly in cases resistant to corticosteroids or when their use is contraindicated. Nevertheless, the authors highlight substantial challenges in interpreting these findings due to considerable heterogeneity among treatment protocols. The comparison and synthesis of results are further complicated by multiple confounding variables, including the type and concentration of the photosensitizing agent, duration of exposure, irradiation parameters, number of treatment sessions, as well as the clinical forms and quantity of OLP lesions evaluated, in addition to variations in study design and methodological rigor.

The most commonly used photosensitizer is 5-aminolevulinic acid (5-ALA) [32,33,34,35,36,37], followed by methylene blue [38,39,40,41,42,43], methyl 5-aminolevulinate (MAL) [44], and chlorin-e6-derivatives [45]. Photosensitizers can be administered as a mouth rinse at a concentration of 1 mg/mL [38,39,40,41,42,43] or applied topically to OLP lesions at concentrations ranging from 5% to 10% [32,33,34,35,36,37,44,45,46]. Studies have evaluated mixed forms of OLP lesions [32,35,38,39,40,41,42,43,44,45], while some investigations have specifically focused on the efficacy of PDT in distinct clinical presentations, such as reticular [34,37] or erosive OLP [33].

Another limitation affecting comparability across studies is the variability in the number of OLP lesions included in the analyses, ranging widely from 10 patients to 144 lesions [32,33,34,35,36,37,38,39,40,41,42,43,44,45,46]. The number of PDT sessions varied between 1 and 10, with intervals of 1–2 weeks between treatments [31,32,33,34,35,36,37,38,39,40,41,42,43,44,45,46,47]. Irradiation parameters also differed considerably, ranging from 7.2 J/cm^2^ [42] to 150 J/cm^2^ [34]. Light sources employed across these studies were diverse, including diode lasers [35,38,39,43], diode lamps [32,33,34,36,37,42,44,46] metal halide lamps [41], semiconductor lasers [45], and GaAlAs lasers [47]. Follow-up periods ranged from 4 weeks to 4 years [38,44].

The efficacy of PDT in the treatment of OLP has been reported with variable outcomes—ranging from no clinical response to complete resolution of lesions and prolonged periods of remission [32,33,34,35,36,37,38,39,40,41,42,43,44,45,46,47]. A systematic review by Akram et al. (2018) [31], which included six clinical studies evaluating the effectiveness of PDT compared to corticosteroid therapy or no treatment, concluded that PDT exerts a measurable therapeutic effect in the management of OLP in adult patients over the course of follow-up. Two of the included studies were randomized controlled trials (RCTs) [43,47], while the remaining four were non-randomized studies [38,40,42,45]. In three studies [42,43,47], local corticosteroid therapy served as the control group. Across all six studies [38,40,42,43,45,47], the follow-up period ranged from 4 to 48 weeks.

Considering its minimally invasive nature, low incidence of adverse effects, and therapeutic efficacy at least comparable to that of corticosteroid therapy, PDT appears to be a promising alternative in the management of OLP—particularly in patients for whom corticosteroid treatment is contraindicated.

Although 5-aminolevulinic acid (5-ALA) is considered a safe and well-tolerated photosensitizer, ensuring its effective retention on the moist oral mucosa remains a significant clinical challenge [33,34,35,36]. To address this issue, the authors described the development of an oromucosal emulgel with enhanced mucoadhesive properties, designed to improve both the local retention and therapeutic efficacy of the photosensitizing agent during treatment [37,48,49]. Among the tested formulations, the TG/XA-based emulgel demonstrated the most favorable combination of mucoadhesion (resulting from hydrogen bonding and electrostatic interactions with mucin chains), release kinetics, and epithelial accumulation, thereby significantly enhancing local ALA retention [48,49].

Clinical observations, including a case series by Sulewska et al. [36], suggest that OLP lesions located on non-keratinized mucosa (e.g., buccal surfaces) respond more favorably to PDT than those on keratinized mucosa (e.g., gingiva) [36,37].

Therefore, the aim of the present study was to evaluate the efficacy of topical corticosteroids and ALA-based photodynamic therapy (ALA-PDT), according to the treatment protocol established by the authors, with particular attention to lesion location within the oral mucosa. The primary objective of the study was to clinically compare the efficacy of topical corticosteroid therapy with photodynamic therapy by evaluating changes in the surface area of OLP lesions (expressed in cm^2^), as well as the severity of OLP manifestations using the REU scoring system (Reticulation, Erythema, Ulceration). The secondary objective was to assess patient satisfaction with the treatment using the Visual Analog Scale (VAS).

## 2. Materials and Methods

### 2.1. Study Characteristics

This study was conducted at the Department of Periodontal and Oral Mucosa Diseases, Medical University of Białystok, between 2020 and 2023. The study was initiated during the pandemic period. Due to limited access to treatment, the number of patients with OLP seeking help was high. Therefore, all patients with a histopathological diagnosis of OLP chose to undergo treatment and participate in the study. Initially, 100 patients were assessed for eligibility; however, 10 individuals declined to participate in the corticosteroid therapy (CT) group—2 during the allocation phase and 8 after completing corticosteroid treatment. Unfortunately, the reasons for this decision are unknown, as patients have the right to withdraw from the study at any stage without providing an explanation. In the photodynamic therapy (PDT) group, all assigned patients completed the full study protocol.

Ultimately, 90 patients (72 women and 18 men), aged 29–88 years (mean age 60 ± 11.7), were enrolled in the study (Table 1 and Table 2).

The sample size was calculated assuming 80% power and a significance level of α = 0.05. For the repeated measures ANOVA test, the required minimum sample size was 28, while for the independent group comparison test, the minimum was 128. At the time of participant inclusion, the final number of lesions, which constitute the study population, was unknown. Therefore, to meet the required minimum sample size, it was decided to include 100 participants in the study, taking into account cases with more than one OLP lesion. Ultimately, the analysis included 90 participants, resulting in 161 lesions (after excluding 10 individuals, including 8 who withdrew from the study). Since the sample size exceeded the calculated minimum, post hoc power analysis was not performed, as it was assumed to be above 80%. The study was approved by the Bioethics Committee of the Medical University of Białystok (approval no. APK.002.372.2021) and conducted in accordance with CONSORT 2025 guidelines. Written informed consent was obtained from all participants.

Inclusion criteria: histologically confirmed OLP diagnosis and age ≥ 18 years. Exclusion criteria were as follows: pregnancy, lactation, systemic diseases (e.g., dermatologic, oncologic, severe liver conditions), known allergies, the use of immunomodulatory/suppressive drugs, antidepressants, or bone anti-metabolites, photosensitivity, allergy to photosensitizer components, recent OLP therapy (<6 months), the inability to attend follow-up visits, or the presence of non-OLP oral lesions.

The clinical examination was conducted by a blinded, calibrated investigator. Calibration was performed on 10 patients not participating in the study. Two measurements were taken 24 h apart. The maximum allowable difference between measurements was 0.5 cm.

Blinding applied only to the investigator performing the clinical examination. The investigator was unaware of the type of medical procedure and was not permitted to ask patients any questions regarding the treatment type. Similarly, patients were instructed not to discuss the type or progress of their treatment with the investigator. However, the study design did not allow for patient blinding, as completely different therapeutic procedures were being compared.

**Figure 1:**
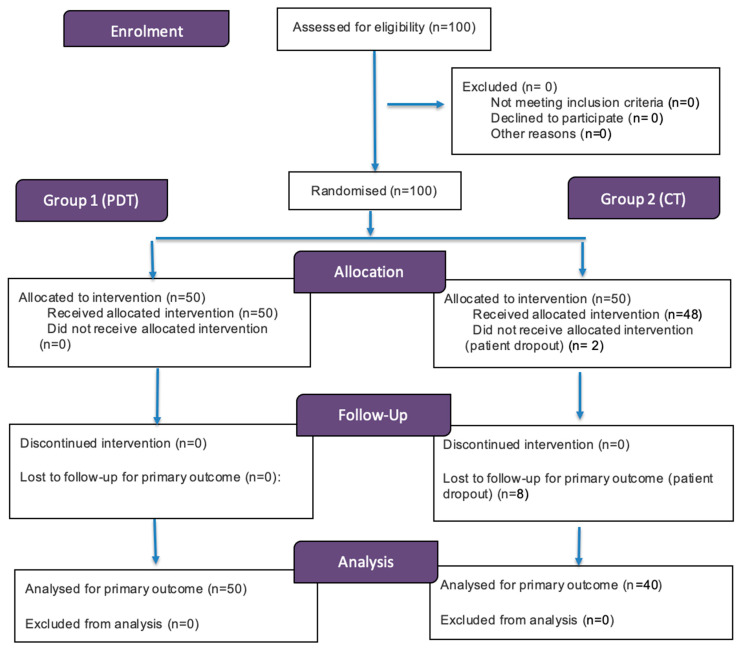
Patient’s flow chart.

This randomized clinical trial allocated patients using a computer-generated randomization list into two groups: PDT-treated Group 1 that received a designed emulgel formulation with photosensitizer 5-ALA at a concentration of 5% (patent P.443813 (PL)), and Group 2 received topical corticosteroid therapy (CT) (Figure 1).

The randomization was prospective and based on the treatment method as the grouping criterion. The location of the lesions was not a criterion for assignment to the randomized subgroup, since a single patient could have multiple OLP lesions located on both keratinized and non-keratinized mucosa. In cases where a patient had multiple OLP lesions, all lesions were treated in the same way according to the assigned group (PDT or CT).

### 2.2. Treatment Protocols

Group 1 (ALA-PDT):

After drying the mucosa, a 2 mm ALA-loaded emulgel was applied to the lesion and surrounding tissue twice, 40 and 20 min before irradiation. The site was covered with an occlusive dressing secured by gauze. Illumination was performed using a FotoSan^®^ 630 LED lamp (CMS Dental, Roslev, Denmark) emitting light at a wavelength of 630 nm, with a power output of 300 mW and an energy density of 108 J/cm^2^. The beam was delivered in non-contact mode at approximately 2 mm from the lesion, applied in a single continuous stage without interruptions for 6 min per square centimeter of each lesion. Five sessions were conducted at weekly intervals.

The applied Fotosan 630 diode lamp emits light within the wavelength range of 620–640 nm, with a distinct peak emission at 630 nm.

No local anesthesia was used prior to the application of the gel in any of the patients.

Group 2 (CT):

Patients applied clobetasol propionate cream (Clobederm 0.5 mg/g; Bausch-Health, Rzeszów, Poland) twice daily for two weeks directly to the lesions.

### 2.3. Clinical Evaluation

Photographic and macroscopic measurements were performed before treatment, immediately after, and six months after treatment. Lesion size was calculated from the maximum length and width using a periodontal probe (PCPUNC 15; Hu-Friedy, Chicago, IL, USA). A calibrated, blinded examiner performed all assessments. Calibration was based on duplicate measurements in 10 non-study patients (acceptable difference < 0.5 cm). Additionally, a digital camera (Nikon D7000, Nikon Corp., Tokyo, Japan) was used for photographic measurements.

The REU index (Reticulation, Erythema, Ulceration) was used to score lesion severity [50]. R was scored as 0 (absent) or 1 (present); E and U were scored as 0 (absent), 1 (<1 cm^2^), 2 (1–3 cm^2^), or 3 (>3 cm^2^). The total REU score was the sum of all components.

Subjective symptoms (pain, burning, itching) were assessed using the Visual Analog Scale (VAS) from 0 (none) to 10 (extreme), with severity classified as follows: 0—none; 1–3—mild; 4–6—moderate; 7–9—severe; 10—extreme [51].

### 2.4. Statistical Analysis

Descriptive statistics included mean (95% CI), standard deviation, median, quartiles, and ranges. The Shapiro–Wilk test assessed normality. Non-normal data were analyzed using the Friedman test and Dunn’s multiple comparisons test (Bonferroni-corrected) or the Mann–Whitney U test. Normally distributed data with sphericity violations were analyzed using MANOVA and Tukey’s HSD. A *p*-value < 0.05 was considered significant. Analyses were performed using Statistica v13 (StatSoft Inc., Tulsa, OK, USA) and PQStat v1.8.2 (PQStat Software, Plewiska, Poland).

## 3. Results

A total of 161 OLP lesions were recorded: 132 on non-keratinized and 29 on keratinized mucosa.

### 3.1. Lesion Dimensions by Treatment and Mucosal Location

Complete resolution occurred in 43.18% (56 lesions) of non-keratinized and 27.58% (8 lesions) of keratinized lesions.

Non-keratinized lesions decreased from 2.54 ± 1.86 cm^2^ pre-treatment to 1.06 cm^2^ post-treatment (a significant reduction of 58.27%) and 1.00 cm^2^ (reduction of 60.63%) at 6-month follow-up (*p* < 0.0001). Keratinized lesions decreased from 1.72 ± 0.82 cm^2^ to 1.12 cm^2^ post-treatment (a reduction in lesion size of 34.88%) but increased to 1.27 cm^2^ at follow-up (26.16%; *p* < 0.0001). Baseline differences between mucosal types were significant (*p* = 0.0207; Mann–Whitney test) (Table 3).

Before treatment, the average surface area of OLP lesions on non-keratinized mucosa was 2.54 cm^2^ ± 1.86. Immediately after the end of treatment, the lesion size significantly decreased by 1.48 cm^2^, and after a 6-month follow-up, by an additional 0.6 cm^2^ (Table 2). In contrast, treatment of OLP lesions on keratinized mucosa was less effective. The average surface area of lesions, initially 1.72 cm^2^ ± 0.82, decreased by 0.62 cm^2^ after treatment, but increased by 0.15 cm^2^ after the 6-month follow-up. Over the 6-month period, the reduction in lesion size on non-keratinized mucosa was 60.63%, whereas on keratinized mucosa it was significantly lower, at 26.16% (Table 3).

On non-keratinized mucosa, PDT (Group 1) treated 72 lesions; CT (Group 2) treated 60. Complete resolution occurred in 61.1% (44 lesions of OLP) in the Group 1 vs. 20% (12 lesions of OLP) in the group 2. Lesion size in the PDT group reduced from 2.64 cm^2^ to 0.56 cm^2^ at follow-up (representing a 78.79% reduction, *p* < 0.0001), while in the CT group it decreased from 2.43 cm^2^ to 1.08 cm^2^ post-treatment, then increased to 1.54 cm^2^ at follow-up (corresponding to a 36.63% overall reduction 36.63%, *p* < 0.0001). Statistically significant differences between the groups were observed only at the 6-month follow-up (*p* < 0.0001).

A total of 132 OLP lesions were located on non-keratinized oral mucosa, of which 72 were treated with photodynamic therapy (PDT) and 60 with corticosteroid therapy. Following PDT, complete remission was observed in 44 cases (61.1%), whereas after corticosteroid therapy, remission was achieved in 12 cases (20%).

Immediately after the completion of PDT, the average size of OLP lesions on non-keratinized mucosa was significantly reduced by 1.59 cm^2^, and by an additional 0.49 cm^2^ at the 6-month follow-up, although this further reduction was not statistically significant.

Corticosteroid therapy also resulted in a significant reduction in lesion surface area—by 1.35 cm^2^ immediately after treatment. However, at the 6-month follow-up, a tendency toward lesion enlargement was observed, although the differences between all time points remained statistically significant.

In keratinized mucosa, 21 OLP lesions were subjected to PDT, while 8 were treated with CT. PDT reduced lesion size from 1.83 to 1.11 cm^2^ (a decrease in OLP lesion size by 39.34%, *p* < 0.0001), while CT reduced it from 1.44 to 1.06 cm^2^, then it rose to 1.69 cm^2^ (an increase in lesion size by 17.36%, *p* = 0.0035). No significant intergroup differences were noted at any time point.

In Group 1, photodynamic therapy (PDT) was applied to 21 OLP lesions, while in Group 2, corticosteroid therapy was used to treat 8 lesions located on keratinized oral mucosa. Complete healing was observed in 7 cases (33.33%) treated with PDT and in 1 case (12.5%) treated with corticosteroids.

Immediately after PDT, the size of OLP lesions on keratinized oral mucosa significantly decreased by 0.69 cm^2^ and remained stable at the 6-month follow-up.

Following corticosteroid therapy, a significant reduction in lesion area by 0.38 cm^2^ was also observed. However, at the 6-month follow-up, the lesions had increased in size by 0.63 cm^2^ compared to the post-treatment measurement, resulting in a significant enlargement of 0.25 cm^2^ relative to the pre-treatment baseline (Table 4).

### 3.2. REU Index

The REU index declined over time, particularly for non-keratinized lesions: from 1.49 ± 0.9 to 0.84 post-treatment and 0.69 at follow-up (*p* < 0.0001). For keratinized mucosa, it decreased from 1.83 ± 0.71 to 1.24 and 1.21, with a significant difference only from baseline to follow-up (*p* = 0.0314) (Table 5).

Before treatment, the mean REU value on non-keratinized oral mucosa was 1.49 ± 0.9. Immediately after treatment, the mean REU significantly decreased by 0.65 and by an additional 0.15 after 6 months of follow-up.

In contrast, the mean REU on keratinized oral mucosa, which was 1.83 ± 0.71 before treatment, decreased non-significantly by 0.59 after treatment, and by a further 0.03 after 6 months. However, the difference between the pre-treatment and 6-month follow-up measurements was statistically significant (Table 5).

By treatment group: PDT reduced REU from 1.65 to 0.68, corresponding to a 58.79% reduction in the severity of OLP lesions, whereas CT reduced it from 1.30 to 0.67 but rose to 0.70 at follow-up (46.15%). Intergroup differences were significant only post treatment (*p* = 0.0014). On keratinized mucosa, REU decreased after PDT (1.86 to 1.10; *p* = 0.0261), while CT showed no significant change (*p* = 0.1462).

Immediately after PDT, the mean REU on non-keratinized oral mucosa significantly decreased by 0.66, and by an additional 0.31 at the 6-month follow-up, although this latter difference was not statistically significant. In the first evaluation after corticosteroid therapy, a significant REU reduction of 0.63 was also observed; however, at the 6-month follow-up, an upward trend was noted, with REU increasing by 0.03.

Immediately after PDT, the mean REU on keratinized oral mucosa decreased non-significantly by 0.53 (28.43%), and by an additional 0.23 (40.32%) at the 6-month follow-up, resulting in a statistically significant difference compared to baseline. In contrast, REU changes over time following corticosteroid therapy were not statistically significant. Although REU decreased by 0.75 (42.86%) immediately after treatment, it increased by 0.5 in the following months, resulting in an overall REU reduction of only 14.29% compared to the pre-treatment lesion severity (Table 6).

### 3.3. Pain Intensity (VAS)

Baseline median VAS scores were 3 (non-keratinized) and 4 (keratinized). VAS scores decreased significantly post treatment and at follow-up (*p* < 0.0001), with changes only significant from baseline to post treatment. At follow-up, VAS ranged from 0 to 4 (non-keratinized) and from 0 to 6 (keratinized) (Table 7).

Before treatment, patients classified pain symptoms as 3 on the VAS for non-keratinized mucosa and 4 for keratinized mucosa. In both groups, the maximum VAS value was 10. After treatment, symptoms decreased over time regardless of the lesion location; however, statistically significant differences in VAS scores were observed only between the measurements taken before treatment and immediately after the end of treatment. The range of minimum and maximum values decreased to 0–4 and 0–6 for non-keratinized and keratinized mucosa, respectively, at the final examination (6 months) (Table 7).

For non-keratinized lesions, the PDT group VAS score decreased from 3.0 to 0.0; the CT group’s score decreased from 3.0 to 1.0, and then increased to 2.0 (a 33% reduction in VAS; *p* < 0.0001). Significant intergroup differences were observed only at follow-up (*p* < 0.0001).

After PDT application, there was a consistent and significant reduction in pain symptoms over the six-month observation period. In this group, patients reported a maximum VAS score of 10 before treatment, compared to the corticosteroid-treated group, in which the maximum pre-treatment VAS score was 7. Although patients in the corticosteroid group reported a faster pain reduction initially, their symptoms were greater during long-term follow-up. Six months after corticosteroid therapy, the maximum VAS score was 4, whereas after PDT it was 3.

For keratinized lesions, the baseline VAS score was 4 in both groups, with higher max scores in PDT patients (10 vs. 6). At follow-up, PDT reduced the VAS score to 1.0 (an average reduction of 75%), while corticosteroid therapy (CT) reduced it to 3.0 (an average reduction of 25%), with no significant improvement from baseline in the CT group. The differences between groups were statistically significant only at follow-up (*p* = 0.023).

Although the median VAS score before treatment was 4 in both groups, patients in Group 1 (PDT) reported the maximum possible VAS value of 10, compared to Group 2, in which the maximum VAS score was 6. Following PDT, symptoms steadily and significantly decreased, whereas in the corticosteroid topical application group, symptom reduction was observed only immediately after treatment. At the 6-month follow-up, patients in Group 1 reported a maximum VAS value of 3, while those in Group 2 reported a value of 6, which was identical to the baseline.

**Figure 2:**
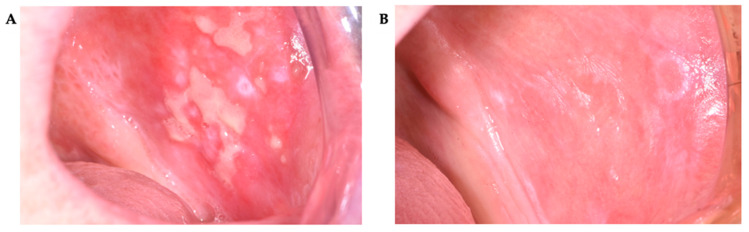
A 65-year-old woman with an OLP lesion on the left buccal mucosa. Post-treatment outcome of OLP lesion on non-keratinized oral mucosa following PDT. (**A**)—baseline condition (T0); (**B**)—six months after completion of treatment (T2) (partial).

**Figure 3:**
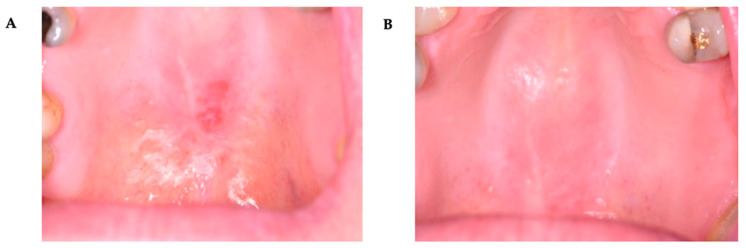
A 74-year-old woman with an OLP lesion on the hard palate. Post-treatment outcome of OLP lesion on keratinized oral mucosa following PDT. (**A**)—baseline condition (T0); (**B**)—six months after completion of treatment (T2) (almost complete).

**Figure 4:**
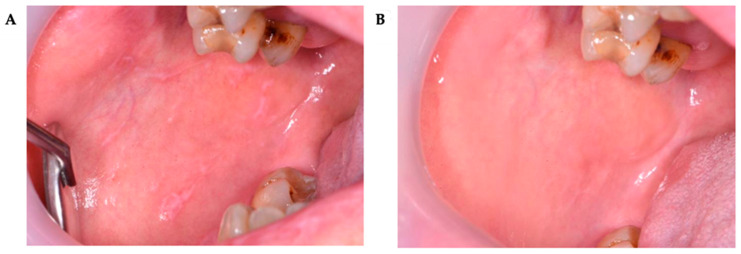
A 52-year-old woman presented with an OLP lesion on the right buccal mucosa. Post-treatment outcome of OLP lesion on non-keratinized oral mucosa following CT. (**A**)—baseline condition (T0); (**B**)—six months after completion of treatment (T2) (almost complete regression of OLP lesions).

**Figure 5:**
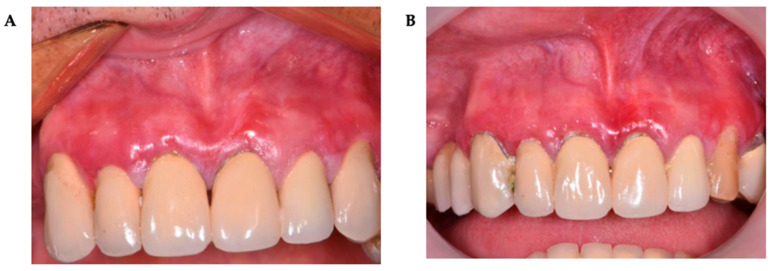
A 62-year-old man presented with an OLP lesion on the gingiva on the maxilla. Post-treatment outcome of OLP lesion on keratinized oral mucosa following CT. (**A**)—baseline condition (T0); (**B**)—six months after completion of treatment (T2) (lack of response to treatment).

These changes were statistically significant over time. Although no differences in VAS scores between Groups 1 and 2 were observed before treatment or immediately after its completion, a significant difference was found at the 6-month post-treatment assessment (Table 8) (Figure 2, Figure 3, Figure 4 and Figure 5).

## 4. Discussion

This study confirms the superior efficacy of ALA-based photodynamic therapy over topical application of corticosteroids in treating oral lichen planus, particularly on non-keratinized mucosa, concerning lesion size, REU score, and pain intensity.

ALA-PDT led to significant, progressive lesion reduction on non-keratinized mucosa (2.64 to 0.56 cm^2^), while CT showed temporary benefit only, with relapse by 6 months (1.08 to 1.54 cm^2^). Both therapies were less effective on keratinized mucosa, with the CT group showing recurrence.

The permeability of the oral mucosa depends on the thickness of the epithelium and the degree of keratinization. The stratum corneum constitutes the main barrier to drug absorption. In cases of mucosal damage (such as erosions or ulcerations), this barrier is disrupted, which increases drug diffusion into the tissues compared to intact epithelial areas [52,53].

Few topical formulations have been specifically developed for the treatment of oral mucosal diseases. Topical drug administration may represent a more targeted and effective option than systemic delivery for oral mucosal conditions. The permeability of topical drugs varies depending on epithelial thickness and keratinization degree. Loss of the permeability barrier in the oral mucosa, caused by ulceration or erosion, leads to faster drug diffusion into tissues compared to unaffected mucosal areas. Each therapy requires distinct drug penetration and retention profiles to optimize treatment efficacy and minimize adverse effects [52,53].

The higher treatment efficacy of OLP lesions located on non-keratinized mucosa can be explained by the phenomenon. In the future, intentional mucosal injury could also be considered to enhance drug penetration (diffusion). Mucosal permeability mainly depends on the thickness and degree of keratinization of the tissues. Their permeability barrier is conditioned by the presence of lipids [54].

We decided to use the ALA emulgel due to its desirable properties in PDT. Mucoadhesive emulgels formulated with anionic natural gums, such as tragacanth (TG) and xanthan (XA), are capable of penetrating the mucin layer and forming hydrogen and electrostatic bonds, resulting in a cohesive film that resists salivary washout. This formulation enables controlled release of 5-aminolevulinic acid (ALA) and the formation of a transient tissue depot, maintaining therapeutic concentrations of the photosensitizer at the application site throughout the irradiation period. Among the tested formulations, the TG/XA-based emulgel demonstrated the most favorable combination of mucoadhesion, release kinetics, and epithelial accumulation, thereby significantly enhancing local ALA retention [48,49].

Carriers of photosensitizers and steroids offer a more predictable method of drug delivery to the mucous membrane. Jurczyszyn et al. [55] demonstrated that, at wavelengths of 405 and 450 nm, pathological lesions in keratinized mucosa exhibit significantly worse textural characteristics compared to lesions in mucosa covered by non-keratinized epithelium [55].

Keratinization of the mucous membrane and the thickness of the stratum corneum act as barriers to the absorption of substances—including photosensitizers. Studies measuring PpIX fluorescence after MAL-PDT application [44] confirm that the photosensitizer penetrates the epithelial and subepithelial layers, indicating that in OLP lesions (which exhibit some degree of keratinization), there is at least partial absorption. However, the data are inconclusive regarding the extent to which keratinization limits this penetration. In the analysis of fractal dimension and autofluorescence, the poorer appearance of lesions in keratinized mucosa at specific wavelengths may indicate reduced light penetration and lower accumulation of fluorescence (i.e., photosensitizer or its active products) in these areas. This could translate to decreased PDT efficacy in keratinized regions [55]. Additionally, general data on mucous membranes suggest that locations with greater keratinization or keratinized structures (e.g., gingiva, hard palate) exhibit lower drug permeability [56].

Similarly, REU analysis demonstrated that ALA-PDT led to sustained reductions in lesion severity of 58.79% (1.65 to 0.68), while CT showed only transient improvement. Only PDT showed significant improvement in keratinized mucosa.

The VAS scores further support PDT’s superiority—the median VAS score dropped to 0.0 on non-keratinized mucosa, while CT improvements were transient, with an overall reduction of 33%. Only PDT showed lasting benefit on keratinized mucosa. For lesions located on keratinized mucosa, PDT demonstrated a 75% reduction in VAS scores whereas CT reduced it to 3.0, corresponding to an average reduction of only 25%, with no statistically significant improvement from baseline in the CT group. These findings are consistent with our previous results [33,34,35,36,37,45].

To our knowledge, no prior randomized clinical trials have compared ALA-PDT to topical CT in OLP treatment. Existing literature consists mainly of case reports or series [34,35,36,57]. Comparisons with other photosensitizers are limited due to heterogeneous methodologies and outcome measures [42,57,58,59,60,61]. Additionally, the lack of standardized protocols and measurement tools poses a challenge and constitutes a limitation of the presented study.

Meta-analyses highlight that PDT efficacy depends on lesion site, size, photosensitizer concentration, contact time, and light wavelength. Intraoral application is complicated by saliva and mucosal movement. High-concentration topical application (e.g., 5% ALA) is more effective than mouth rinses [30,62,63]. Most commercial formulations lack adequate ability to adhere to mucosal tissue. Designed emulgel, based on tragacanth and xanthan gum, offers beneficial mucoadhesive properties and enhanced ALA retention at the application site [48,49].

These findings are consistent with prior studies showing greater pain reduction with PDT than CT [31,43,63,64], particularly in long-term follow-up [36,44,65]. PDT is generally safe, with transient adverse effects such as erythema, edema, or pain during irradiation [23,58], typically resolving within 24 h. In the present study, six patients reported mild discomfort, none of whom required intervention.

In the presented study, lesions located on keratinized oral mucosa showed a lower healing rate than those on non-keratinized mucosa. Previously published by our group data revealed that the designed emulgel enhanced drug permeability and accumulation in non-keratinized epithelium [48]. Lower therapeutic effect of ALA-PDT might be due to the lower permeability rate of the photosensitizer through the keratinized layer, as demonstrated by Selvam et al. [66] and Yan et al. [67]. In turn, in the CT group, the reduced efficacy may result from accumulating the drug in the superficial epithelial layer, with the thick keratin barrier preventing deeper penetration into the lesion [68,69]. Additionally, the superior outcomes observed in the PDT-treated group may be partially attributed to the mucoadhesiveness of the emulgel formulation that favored drug retention at the application site, though the supportive effect of occlusive dressing during photosensitizer application cannot be excluded. In contrast, patients treated with corticosteroids were instructed to self-apply the topical ointment product without the ability to adhere to the oral mucosa. Moreover, PDT may also provide additional benefit by better reducing oxidative stress—one of the key etiological factors in oral lichen planus—thereby addressing not only symptoms but also underlying pathogenic mechanisms [70]. It should also be noted that, CT may induce candidiasis, mucosal atrophy, and systemic effects [71]. Although only unpleasant taste was reported in the tested cohort, such effects may be clinically significant in older adults requiring prolonged therapy.

One limitation of the study is the relatively short, 6-month follow-up period, which, in the case of a chronic condition such as OLP, requires extension to better capture long-term outcomes. Another limitation concerns the heterogeneity of the analyzed subgroups—particularly the small number of lesions located on keratinized mucosa. A further constraint is the potential subjectivity of lesion size measurements, due to the inherent challenges of performing measurements within the oral cavity. These include the curvature of anatomical structures, the presence of muscular tissue—which allows for surface stretching—and the continuous flow of saliva.

The assessment of oxidative stress markers was the subject of a separate investigation, the results of which were published earlier this year [70].

### Side Effects

PDT is considered a safe method, with adverse effects including skin reactions such as erythema and swelling [56,72,73], as well as contact dermatitis at the site of photosensitizer application [74]. However, the most common side effect is burning or pain at the irradiation site, which may require additional analgesic treatment [33,34]. Our previous studies have detailed the severity of discomfort following ALA-PDT sessions, reporting burning or pain in patients with both erosive and reticular forms of OLP. Although some patients reported moderate to severe pain and burning sensations post-irradiation (on a scale from 0 to 3), in most cases the discomfort resolved by the next day and did not necessitate analgesic use [71].

In the present study, six patients (12%) in the PDT group reported mild mucosal burning or pain during irradiation, which resolved no later than two days after the session. In the corticosteroid therapy (CT) group, 10 patients (25%) reported mild discomfort in the form of an unpleasant taste. Although most reported adverse effects were mild, their impact on the patient should not be underestimated, especially considering the chronic nature of the disease and the fact that it affects elderly patients.

Topical corticosteroid use can lead to candidiasis, mucosal atrophy, dry mouth, burning sensations, or gastrointestinal disturbances [38]. Therefore, in the context of OLP patients who may be more sensitive to drug side effects, the choice between photodynamic therapy and corticosteroids becomes crucial.

## 5. Conclusions

Although the effectiveness of PDT in the treatment of OLP—particularly on non-keratinized oral mucosa—appears promising, the results should be interpreted with caution due to the need for long-term, multicenter, randomized clinical trials before its widespread implementation in OLP therapy.

Attention should also be paid to the need for then standardization and development of a unified research protocol to enable meaningful comparisons between studies.

## 6. Patents

Szymańska et al. [48,49], A pharmaceutical composition with mucoadhesive properties and its use. P.443813 (PL); PCT/IB2024/051420—PCT application number.

## Figures and Tables

**Table 1:** Demographic and clinical characteristics of study participants.

Variable	N (%)
Gender	
Male	18 (20%)
Female	72 (80%)
Age	
Average age (M ± SD)	60 ± 11.7 years
Lesion location	
Non-keratinized mucosa	132 (81.99%)
Keratinized mucosa	29 (18.01%)

**Table 2:** Range of age distribution of patients in PDT and CT groups.

Group	Sex	Age < 40	Age ≥ 40 ≤ 65	Age > 65	Total
PDT	M	0	3	4	7
	F	5	23	15	43
CT	M	3	4	4	11
	F	0	18	11	29
Total		8	48	34	90

**Table 3:** Mean, standard deviation, confidence interval, median, and minimum and maximum values of the size of OLP lesions located on non-keratinized (NK) and keratinized (K) oral mucosa before treatment (T0), immediately after treatment (T1), and six months after treatment (T2).

Time Point	Type of Mucosa.	Mean	Standard Deviation	95% CI (Min)	95% CI (Max)	Median	Minimum	Maximum	*p* (Friedman Test)
T0	NK	2.54	1.86	2.22	2.86	2.25	0.15	12.00	<0.0001
T1		1.06	1.38	0.83	1.30	1.00	0.00	10.00	
T2		1.00	1.59	0.73	1.28	0.50	0.00	12.00	
T0	K	1.72	0.82	1.41	2.04	1.50	0.72	4.00	<0.0001
T1		1.12	1.03	0.73	1.51	1.00	0.00	4.00	
T2		1.27	1.36	0.76	1.79	0.75	0.00	4.00	

**Table 4:** Mean, standard deviation, confidence interval, median, minimum, and maximum values of OLP lesion size on keratinized and non-keratinized oral mucosa in groups 1 (PDT) and 2 (CT), measured before treatment (T0), immediately after treatment (T1), and 6 months post treatment (T2).

Location	Group	Time Point	Mean	SD	95% CI (Min)	95% CI (Max)	Median	Min	Max	*p* (Friedman Test)
Non-keratinized mucosa	1	T0	2.64	1.75	2.23	3.05	2.25	0.15	9.0	<0.0001
		T1	1.05	0.99	0.81	1.28	1.0	0.0	4.0	
		T2	0.56	0.92	0.34	0.78	0.0	0.0	3.0	
	2	T0	2.43	2.0	1.91	2.94	2.25	0.35	12.0	<0.0001
		T1	1.08	1.74	0.63	1.53	0.55	0.0	10.0	
		T2	1.54	2.02	1.02	2.06	1.0	0.0	12.0	
Keratinized mucosa	1	T0	1.83	0.84	1.45	2.21	1.5	0.75	4.0	<0.0001
		T1	1.14	1.15	0.62	1.67	1.0	0.0	4.0	
		T2	1.11	1.34	0.5	1.72	0.5	0.0	4.0	
	2	T0	1.44	0.76	0.8	2.08	1.25	0.72	2.5	0.0035
		T1	1.06	0.66	0.51	1.61	0.88	0.4	2.0	
		T2	1.69	1.4	0.52	2.86	1.25	0.0	3.75	

**Table 5:** The mean, standard deviation, confidence interval, median, as well as minimum and maximum REU values for lesions located on non-keratinized (NK) and keratinized (K) oral mucosa before treatment (T0), immediately after treatment (T1), and 6 months post treatment (T2).

Time Point	Type of Mucosa	Mean	Standard Deviation	95% CI (Min)	95% CI (Max)	Median	Minimum	Maximum	*p* (Friedman Test)
T0	NK	1.49	0.90	1.34	1.65	1.00	1.00	5.00	<0.0001
T1		0.84	0.58	0.74	0.94	1.00	0.00	2.00	
T2		0.69	0.67	0.57	0.80	1.00	0.00	3.00	
T0	K	1.83	0.71	1.56	2.10	2.00	1.00	3.00	<0.0006
T1		1.24	0.91	0.89	1.59	1.00	0.00	5.00	
T2		1.21	1.05	0.81	1.61	1.00	0.00	5.00	

**Table 6:** Mean, standard deviation, confidence interval, median, minimum, and maximum REU scores for lesions on keratinized and non-keratinized oral mucosa in groups 1 (PDT) and 2 (CT), assessed before treatment (T0), immediately after treatment (T1), and 6 months later (T2).

Location	Group	Time Point	Mean	SD	95% CI (Min)	95% CI (Max)	Median	Min	Max	*p* (Friedman Test)
Non-keratinized mucosa	1	T0	1.65	1.08	1.4	1.91	1.0	1.0	5.0	<0.0001
		T1	0.99	0.54	0.86	1.11	1.0	0.0	2.0	
		T2	0.68	0.73	0.51	0.85	1.0	0.0	3.0	
	2	T0	1.3	0.56	1.15	1.45	1.0	1.0	3.0	<0.0001
		T1	0.67	0.57	0.52	0.81	1.0	0.0	2.0	
		T2	0.7	0.59	0.55	0.85	1.0	0.0	3.0	
Keratinized mucosa	1	T0	1.86	0.73	1.53	2.19	2.0	1.0	3.0	0.0014
		T1	1.33	0.97	0.89	1.77	1.0	0.0	5.0	
		T2	1.1	1.14	0.58	1.61	1.0	0.0	5.0	
	2	T0	1.75	0.71	1.16	2.34	2.0	1.0	3.0	0.1462
		T1	1.0	0.76	0.37	1.63	1.0	0.0	2.0	
		T2	1.5	0.76	0.87	2.13	1.0	1.0	3.0	

**Table 7:** Median, minimum and maximum VAS values, and lower and upper quartiles in the studied group of patients depending on the location of OLP lesions on non-keratinized (NK) and keratinized (K) oral mucosa before treatment (T0), immediately after treatment (T1), and six months after treatment (T2).

Time Point	Type of Mucosa	Median	Minimum	Maximum	Q1	Q3	*p* (Friedman Test)
T0	NK	3.00	2.00	10.00	3.00	4.00	<0.0001
T1		1.50	0.00	4.00	1.00	2.00	
T2		1.00	0.00	4.00	0.00	2.00	
T0	K	4.00	2.00	10.00	3.00	5.00	<0.0001
T1		2.00	0.00	5.00	1.00	3.00	
T2		2.00	0.00	6.00	1.00	2.00	

**Table 8:** Median, minimum, maximum, lower, and upper quartile values of VAS scores in patients with lesions on keratinized and non-keratinized oral mucosa in groups 1 (PDT) and 2 (CT), assessed before treatment (T0), immediately after treatment (T1), and 6 months later (T2).

Location	Group	Time Point	Median	Min	Max	Q1	Q3	*p* (Friedman Test)
Non-keratinized mucosa	1	T0	3.0	2.0	10.0	3.0	4.5	<0.0001
		T1	2.0	0.0	4.0	1.0	2.0	
		T2	0.0	0.0	3.0	0.0	1.0	
	2	T0	3.0	2.0	7.0	3.0	4.0	<0.0001
		T1	1.0	0.0	4.0	0.0	2.0	
		T2	2.0	0.0	4.0	1.0	3.0	
Keratinized mucosa	1	T0	4.0	3.0	10.0	3.0	5.0	<0.0001
		T1	2.0	0.0	3.0	1.0	3.0	
		T2	1.0	0.0	3.0	0.0	2.0	
	2	T0	4.0	2.0	6.0	3.0	5.5	0.0018
		T1	2.0	0.0	5.0	1.0	3.0	
		T2	3.0	0.0	6.0	1.5	4.0	

## Data Availability

Data available on request due to restrictions privacy or ethical reasons.
